# Reach of Supplemental Nutrition Assistance Program–Education (SNAP–Ed) Interventions and Nutrition and Physical Activity-Related Outcomes, California, 2011–2012

**DOI:** 10.5888/pcd12.140449

**Published:** 2015-03-12

**Authors:** Fred Molitor, Sharon Sugerman, Hongjian Yu, Michael Biehl, May Aydin, Melanie Levy, Ninez A. Ponce

**Affiliations:** Author Affiliations: Sharon Sugerman, Michael Biehl, the Nutrition Education and Obesity Prevention Branch, California Department of Public Health, Sacramento, California; Hongjian Yu, May Aydin, Melanie Levy, Ninez A. Ponce, UCLA Center for Health Policy Research, Los Angeles, California.

## Abstract

**Introduction:**

This study combined information on the interventions of the US Department of Agriculture’s Supplemental Nutrition Assistance Program–Education with 5,927 interview responses from the California Health Interview Survey to investigate associations between levels of intervention reach in low-income census tracts in California and self-reported physical activity and consumption of fruits and vegetables, fast food, and sugar-sweetened beverages.

**Methods:**

We determined 4 levels of intervention reach (low reach, moderate reach, high reach, and no intervention) across 1,273 program-eligible census tracts from data on actual and eligible number of intervention participants. The locations of California Health Interview Survey respondents were geocoded and linked with program data. Regression analyses included measures for sex, age, race/ethnicity, and education.

**Results:**

Adults and children from high-reach census tracts reported eating more fruits and vegetables than adults and children from no-intervention census tracts. Adults from census tracts with low, moderate, or high levels of reach reported eating fast food less often than adults from no-intervention census tracts. Teenagers from low-reach census tracts reported more physical activity than teenagers in no-intervention census tracts.

**Conclusion:**

The greatest concentration of Supplemental Nutrition Assistance Program–Education interventions was associated with adults and children eating more fruits and vegetables and adults eating fast food less frequently. These findings demonstrate the potential impact of such interventions as implemented by numerous organizations with diverse populations; these interventions can play an important role in addressing the obesity epidemic in the United States. Limitations of this study include the absence of measures of exposure to the intervention at the individual level and low statistical power for the teenager sample.

## Introduction

Obesity is a precursor to numerous chronic diseases, including coronary heart disease, diabetes, hypertension, some cancers, gall bladder disease, osteoarthritis, sleep apnea, and respiratory problems (1). In the United States, in 2011–2012, the prevalence of adult obesity was 34.9%, more than double that of 1980 (2,3). Also during 2011–2012, the prevalence was 16.9% among children and adolescents aged 2 to 19 years, tripling during the same period (4). Obesity rates have plateaued, showing no significant change for adults or youths between 2003 and 2012 (2).

The US Department of Agriculture (USDA) promotes behaviors that can reduce the impact of the obesity epidemic among low-income populations through its Supplemental Nutrition Assistance Program–Education (SNAP–Ed) program, which is designed to increase the nutritious food choices of, and physical activity by, more than 46 million SNAP participants and people eligible for SNAP (5).

Peer reviewed studies demonstrating that SNAP–Ed improves desired behaviors are limited (6–8). USDA demonstration projects have found significant increases in fruit and vegetable consumption among children and seniors (9). Although the results look promising, these projects should be interpreted within the context that follow-up measures were taken right after the interventions ended; they were high-dose, direct-interaction interventions not representative of the diversity of SNAP–Ed interventions approved and promoted by the USDA; and they included randomized or quasiexperimental designs that are difficult to carry out under the usual conditions of program delivery.

The aim of our study was to test the external validity of SNAP–Ed interventions as implemented by various organizations with diverse populations in California by using an ecological approach. Specifically, using survey data from a random sample of SNAP–Ed-eligible individuals, we assessed by census tract whether level of SNAP–Ed intervention reach was associated with increased consumption of fruits and vegetables, less frequent consumption of fast food and sugar-sweetened beverages, and more physical activity.

## Methods

This study was reviewed and approved by the California Health and Human Services Agency, Committee for the Protection of Human Subjects.

### Study population

The study population was SNAP–Ed-eligible Californians. The Healthy, Hunger-Free Kids Act of 2010 defines SNAP–Ed eligibility at the household and population level. In federal fiscal year (FFY) 2011, SNAP–Ed-eligible people were those in households with income at or below 185% of the federal poverty level (FPL) and people in locations such as schools or geographic areas (eg, census tracts) where 50% or more of the population resided in households at or below 185% of the FPL.

We used 2 databases of SNAP-eligible people. One database included data on people documented as participating in a SNAP–Ed intervention (and thereby SNAP–Ed-eligible) in FFY 2011. The second database included data on participants in the California Health Interview Survey (CHIS) in 2011–2012 who met the household income criterion for SNAP–Ed. Individual responses to selected items on the CHIS questionnaire were linked with estimates of the reach of the SNAP–Ed intervention, calculated by census tract. We used an ecological approach because the CHIS questionnaire did not ask about participating in a SNAP–Ed intervention. Relying on self-reports would have resulted in an invalid operationalization of SNAP–Ed participation and exposure. Most SNAP–Ed contractors do not explicitly identify their interventions as SNAP–Ed or USDA funded, and such questions would be subject to recall and social desirability biases.

### Independent variable

The independent variable, intervention reach, was defined as the number of SNAP–Ed participants divided by the number of SNAP–Ed-eligible people in each California census tract that met the population eligibility requirements for SNAP–Ed in FFY 2011. The number of SNAP–Ed-eligible people in SNAP–Ed-eligible census tracts was determined by using standardized procedures implemented annually by the Nutrition Education and Obesity Prevention Branch (NEOPB) of the California Department of Public Health and is based on data from the US Census and the American Community Survey.

The numerator used to calculate intervention reach (the number of participants in SNAP–Ed interventions in FFY 2011) was obtained from the USDA’s Education and Administrative Reporting System (EARS). In FFY 2011, EARS data were available from 120 NEOPB-funded contractors. NEOPB staff train and provide technical assistance to contractors on collecting and entering data, and they clean and summarize data for program planning and annual reports to the USDA.

Both unduplicated and duplicated counts of SNAP–Ed participants are entered by contractors into EARS for direct education (eg, structured learning interventions facilitated by a trained educator or through interactive media) and indirect education (eg, interventions involving the distribution of information and resources to groups of people in settings such as community fairs and cooking demonstrations). Data on the same individuals can appear in EARS for school-based interventions consisting of multisession classes. Different contractors could also record separately the same individuals for 1 SNAP–Ed intervention.

To eliminate the possibility of duplicate counts of people who participated in a SNAP–Ed intervention with multiple sessions, we counted people in the first session only. Interventions were also sorted by site name in cities, and duplicates were deleted. These procedures resulted in 6.6 million presumed unduplicated individuals who participated in a SNAP–Ed intervention in FFY 2011.

Intervention reach was calculated at the census-tract level by geocoding (ArcGIS version 10.1; Esri) the address of each SNAP-Ed intervention site for each unduplicated SNAP–Ed participant.

### Dependent variables

Data related to 3 intended SNAP–Ed outcomes (healthful eating, healthful beverage consumption, and recommended levels of physical activity) came from the 2011–2012 CHIS, conducted from June 2011 through December 2012 (10).

CHIS is an ongoing stratified random-digit–dialed health survey. Interviews in 2011–2012 were conducted in English, Spanish, Cantonese, Mandarin, Korean, and Vietnamese. Adults, teenagers, or children were randomly selected from each sampled household. Teenagers were interviewed after permission was obtained from their parent or legal guardian, who may or may not have been the adult respondent for the household. Interview data for children were provided by the adult that was identified as most knowledgeable about the child’s health.

The dependent variables for this study were fruit and vegetable consumption, fast food consumption, sugar-sweetened beverage consumption, and physical activity. Proxy questions related to consumption behaviors excluded children younger than 2 years; physical activity questions pertained to children 5 years or older.

Answers to CHIS questions about eating fruit were combined with questions about eating vegetables to develop 1 variable on fruit and vegetable consumption for adults, teenagers, and children. The questions asked of adults were “During the past month, how many times did you eat fruit? Do not count juices.” and “During the past month, how many times did you eat any other vegetables like green salad, green beans, or potatoes? Do not include fried potatoes.” Reported number of times were recorded as “per day,” “per week,” or “per month” on the basis of how respondents chose to answer the questions, and values were summed and converted to a per-day unit.

For teenagers, the open-ended responses to the questions “Yesterday, how many servings of fruit, such as an apple or banana, did you eat?” and “Yesterday, how many servings of other vegetables like green salad, green beans, or potatoes did you have? Do not include fried potatoes.” were combined. Child proxy interviews with adults included the questions “Yesterday, how many servings of fruit, such as an apple or a banana, did (child) eat?” and “Yesterday, how many servings of other vegetables like green salad, green beans, or potatoes did (child) have? Do not include fried potatoes.”

The same question was used to assess fast food consumption among adults, teenagers, and children: “Now think about the past week. In the past 7 days, how many times did you (“he/she” for children) eat fast food? Include fast food meals eaten at work, at home, or at fast-food restaurants, carryout or drive through.” The number of times was recorded.

The types of sugar-sweetened beverages on the market today include regular (nondiet) soda, sweetened fruit drinks, and sports and energy drinks. The 2011–2012 CHIS survey of adults focused on consumption of regular sodas only: “During the past month, how often did you drink regular soda or pop that contains sugar? Do not include diet soda.” Responses were converted to a per-week basis. Interviewers clarified with respondents whether their answers were based on per day, per week, or per month.

The answers to the following 2 open-ended questions for teenagers were combined to assess consumption of sugar-sweetened beverages: “Yesterday, how many glasses or cans of soda that contain sugar, such as Coke, did you drink? Do not include diet soda.” and “Yesterday, how many glasses or cans of sweetened fruit drinks, sports, or energy drinks, did you drink?” The following question was asked to assess consumption among children: “Yesterday, how many glasses or cans of soda, such as Coke, or other sweetened drinks, such as fruit punch or sports drinks did (he/she) drink? Do not count diet drinks.”

Physical activity was measured differently for adults than for teenagers and children. Minutes of walking per week for adults was assessed with a series of questions that asked about number of times per week and number of minutes per day of walking for transportation versus relaxation or exercise. Respondents answered these questions with number of minutes or hours.

Physical activity for teenagers was assessed with the open-ended question “Not including school PE, in the past 7 days, on how many days were you physically active for at least 60 minutes total per day?” Proxy interviews for children included the similar question “Not including school PE, on how many days of the past 7 days was (child) physically active for at least 60 minutes total?”

### Sociodemographic variables

Age was assessed on CHIS through reported date of birth or, if respondent refused, a categorical variable. Adult participants, parents or legal guardians of teenagers, and the “most knowledgeable adult” for children were asked to indicate the highest school grade completed. Sex and race/ethnicity were determined through self-report. FPLs were calculated from responses to questions about household income and number of persons in the household supported by this income.

### Analysis

Data on CHIS participants with an FPL greater than 185% were excluded. The physical addresses of the remaining SNAP–Ed eligible CHIS respondents were geocoded to a census tract and coded for level of intervention reach so that the record for each adult, teenager, and child included a variable (ranging from 0 to 1) that represented the proportion of intervention reach in the census tract where he or she lived.

A dichotomous variable of intervention reach was created to examine (using χ^2^ tests) whether age, sex, education, race/ethnicity, or FPL significantly differed between census tracts that had an intervention and census tracts that had no intervention.

All dependent variables were examined for outliers. Responses of more than 750 minutes of walking per week (among adults) were removed. The distribution of this variable was also skewed to the right (skewness = 4.53). A Box–Cox transformation was performed, and a log transformation was determined to be the most appropriate method for obtaining a normal distribution.

Regression analyses (SAS version 9.3; SAS Institute Inc) were used to examine the relationships between intervention reach and the dependent variables. For these analyses, we established 4 categories of reach based on the distribution of the proportions: 1) no SNAP–Ed interventions; 2) low reach (0.01%–39.99% of the target population reached); 3) moderate reach (40%–89.99%); and 4) high reach (90%–100%). These models controlled for age, sex, education, and race/ethnicity. Data on age were categorized into 7 groups (0–4 y, 5–11 y, 12–17 y, 18–24 y, 25–44 y, 45–64 y, and ≥65 y). Educational attainment was recoded as less than high school or high school or more.

Negative binomial models were developed for outcomes based on counts (fruit and vegetable consumption, fast food consumption, sugar-sweetened beverage consumption, and physical activity for teenagers and children). Linear modeling (ordinary least squares) was used for the continuous outcome of physical activity (minutes per week of walking) among adults.

The models took the following forms: log*it*(μ) = α + *X*β + *CT* (negative binomial model for count outcomes) and *Y* = α + *X*β + *CT* (linear model for continuous outcomes), where in both models, α is the intercept; *X* is the design matrix of the adjusted characteristics age, sex, race/ethnicity, and education; and β is a vector of the regression coefficients associated with those confounders. *C* is a set of indicators for levels of intervention reach; the reference level is the comparison group (no intervention). *T* is the regression coefficient of the intervention reach. For goodness of fit for the linear models, normality of the residual distributions was checked through Q–Q plots and scatter plots.

We hypothesized that SNAP–Ed interventions have a positive impact on the targeted population, and therefore a 1-sided *P* value was selected to determine significance at the .05 level.

## Results

### Characteristics of the sample

In 1,273 SNAP–Ed-eligible census tracts, CHIS interview data were available for 4,245 adults, 465 teenagers, and 1,217 children. The proportion of the sample by level of intervention reach was similar among the 3 age groups: roughly 34% to 36% in the no-intervention group, 36% to 40% in the low-reach group, 10% to 13% in the moderate-reach group, and 15% to 17% in the high-reach group (Table 1).

**Table 1:** 2011–2012 California Health Interview Survey Participants by Age Group and Levels^a^ of Intervention Reach for California Supplemental Nutrition Assistance Program–Education (SNAP–Ed) Among SNAP–Ed-Eligible Census Tracts, Federal Fiscal Year 2011^b^

Age Group	No Intervention (n = 529)	Low Reach (n = 401)	Moderate Reach (n = 134)	High Reach (n = 209)	Total (n = 1,273)
Adults aged ≥18 y	1,507 (35.5)	1,522 (35.8)	482 (11.4)	734 (17.3)	4,245 (100)
Teenagers aged 12–17 y	160 (34.4)	185 (39.8)	48 (10.3)	72 (15.5)	465 (100)
Children aged 2–11 y	409 (33.6)	465 (38.2)	156 (12.8)	187 (15.4)	1,217 (100)

a Levels of reach defined as low (0.01%–39.99% of the target population reached); moderate (40%–89.99% reached); high (90%–100% reached).

b All values are number (percentage).

Overall, 59.6% of adults and most teenagers (87.1%) and children (83.6%) were Hispanic. Two-fifths (40.4%) of adults had less than a high school education, and 56.4% had an FPL of less than 100%. We found no significant differences between the intervention and no-intervention census tracts for age, sex, education, race/ethnicity, or FPL (Table 2).

**Table 2:** Characteristics of 2011–2012 California Health Interview Survey Participants by Age Group and SNAP–Ed-Eligible Census Tracts With and Without SNAP–Ed Interventions, Federal Fiscal Year 2011^a^

Characteristic	Census Tracts That Had Interventions (n = 744)			Census Tracts That Had No Interventions (n = 529)		
	Adults Aged ≥18 y	Teenagers Aged 12–17 y	Children Aged 2–11 y	Adults Aged ≥18 y	Teenagers Aged 12–17 y	Children Aged 2–11 y
**Age, no. (mean, y)**	2,738 (49.4)	305 (14.4)	808 (5.5)	1,507 (49.3)	160 (14.4)	409 (5.7)
**Sex**						
Male	1,043 (38.1)	148 (48.5)	451 (55.8)	560 (37.2)	82 (51.2)	222 (54.3)
Female	1,695 (61.9)	157 (51.5)	357 (44.2)	947 (62.8)	78 (48.8)	187 (45.7)
**Education^b^**						
<High school	1,123 (41.0)	182 (59.7)	410 (50.7)	591 (39.2)	87 (54.4)	200 (48.9)
≥High school	1,615 (59.0)	123 (40.3)	398 (49.3)	916 (60.8)	73 (45.6)	209 (51.1
**Race/ethnicity**						
Hispanic	1,609 (58.8)	260 (85.2)	666 (82.4)	919 (61.0)	145 (90.6)	351 (85.8)
White	558 (20.4)	21 (6.9)	62 (7.7)	262 (17.4)	2 (1.2)	17 (4.2)
Asian	244 (8.9)	12 (3.9)	33 (4.1)	192 (12.7)	4 (2.5)	18 (4.4)
African American	206 (7.5)	6 (2.0)	22 (2.7)	98 (6.5)	7 (4.4)	13 (3.2)
Other race	121 (4.4)	6 (2.0)	25 (3.1)	36 (2.4)	2 (1.2)	10 (2.4)
**Federal poverty level^b^**						
0%–99%	1,561 (57.0)	184 (60.3)	476 (58.9)	835 (55.4)	99 (61.9)	250 (61.1)
100%–185%	1,177 (43.0)	121 (39.7)	332 (41.1)	835 (44.6)	61 (38.1)	159 (38.9)

Abbreviation: SNAP–Ed, Supplemental Nutrition Assistance Program–Education.

a All values are number (percentage) unless otherwise indicated.

b Assigned for teenagers based on consent of parent or legal guardian; assigned for children based on adult identified as most knowledgeable about the child’s health.

Higher levels of intervention reach were related to more healthful eating behaviors among adults (Table 3). Adults from high-reach census tracts ate fruits and vegetables more often than adults in low-, moderate-, or no-reach census tracts. Adults from census tracts with low, moderate, and high levels of reach ate fast food less often than adults in no-intervention census tracts. Contrary to expectations, teenagers living in census tracts with SNAP–Ed interventions ate fast food more often than those from no-intervention census tracts.

**Table 3:** Relationships Between Reach of California SNAP–Ed Interventions in Federal Fiscal Year 2011 and Healthful Eating Behaviors, Consumption of Sugar-Sweetened Beverages, and Physical Activity of Adults, Teenagers, and Children Participating in the 2011–2012 California Health Interview Survey

Age Group/Level of Reach^a^		Fruit and Vegetable Consumption	Fast Food Consumption	Consumption of Sugar-Sweetened Beverages	Physical Activity
Adults Aged ≥18 y	No.	Times Per Day, *z* Value^b^	Times Past Week, *z* Value^b^	Regular Soda, Times Per Week, *z* Value^b^	Walking, Minutes per Week, *t* Value^c^
No intervention	1,507	—	—	—	—
Low	1,522	0.85	−1.67^d^	−0.14	−0.53
Moderate	482	0.39	−2.13^d^	0.40	−1.57
High	734	1.79^d^	−2.08^d^	−1.15	1.05
**Teenagers Aged 12–17 y**	**No.**	**Servings Yesterday, *z* Value^b^**	**Times Past Week, *z* Value^b^**	**Regular Soda, Fruit, Sports, or Energy Drinks, No. of Glasses/Cans Yesterday, *z* Value^b^**	**Physically Active ≥60 Minutes, Days Last Week, *z* Value^b^**
No intervention	160	—	—	—	—
Low	185	−1.14	2.78^e^	1.00	1.81^d^
Moderate	48	−1.26	2.44^e^	0.39	1.26
High	72	−0.55	3.28^e^	1.05	1.13
**Children^f^**	**No.**	**Servings Yesterday, *z* Value^b^**	**Times Past Week, *z* Value^b^**	**Regular Soda, Fruit, Sports, or Energy Drinks, No. of Glasses/Cans Yesterday, *z* Value^b^**	**Physically Active ≥60 Minutes, Days Last Week, *z* Value^b^**
No intervention	409	—	—	—	—
Low	465	0.60	0.07	0.65	0.15
Moderate	156	1.08	−0.15	−0.25	−0.14
High	187	2.07^d^	0.04	−0.44	1.47

Abbreviation: SNAP–Ed, Supplemental Nutrition Assistance Program–Education.

a Levels of reach defined as low (0.01%–39.99% of the target population reached); moderate (40%–89.99% reached); high (90%–100% reached).

b Negative binomial regression analyses controlling for sex, race/ethnicity, education, and age for adults (18–24 y, 25–44 y, 45–64 y, and ≥65 y).

c Linear regression analyses controlling for sex, race/ethnicity, education, and age for adults (18–24 y, 25–44 y, 45–64 y, and ≥65 y).

d *P* < .05, 1-sided, based on hypothesized direction.

e *P* < .05, 2-sided, based on nonhypothesized direction.

f Consumption questions asked about children aged 2 through 11 years; physical activity questions were answered by most knowledgeable adults of children 5 through 11 years.

Children from high-reach census tracts ate more fruits and vegetables than children from no-intervention census tracts. Levels of intervention reach were not related to levels of consumption of sugar-sweetened beverages in any of the 3 age groups.

Teenagers from low-reach census tracts reported more physical activity than teenagers in no-reach census tracts. 

## Discussion

The greatest concentration of SNAP–Ed interventions was related to eating more fruits and vegetables among adults and children and eating fast food less often among adults only. The finding of increased fruit and vegetable consumption among children and adults confirms the findings of USDA demonstration projects designed with an emphasis on internal validity (9). Our study results suggest that such outcomes are generalizable to low-income people throughout California and that different types of SNAP–Ed interventions implemented by different types of organizations in diverse populations can lead to greater intake of fruits and vegetables. Novel to the scant research on the positive impacts of SNAP–Ed was our finding that adults from SNAP–Ed areas improved dietary behaviors by eating fast food less often.

SNAP–Ed interventions include messages to adults on the health benefits of fruits and vegetables and preparing meals at home, the provision of healthful recipes, and demonstrations on how to prepare fruits and vegetables. These educational messages and newly learned skills may have been responsible for changes to the snacks and meals made and eaten by parents at home, which in turn translated into increased fruit and vegetable consumption by their children. Many FFY 2011 interventions were school-based, directly targeting children. Lower levels of fast food consumption among adults may be explained by behavior changes during the day, when parents were more likely to rely on the convenience of fast food. SNAP–Ed interventions may have prompted parents to alter their choices away from fast food when out of the house for work or errands, for example, while their children were attending day care or school.

Contrary to our hypotheses, we found that census tracts with higher reach were associated with greater fast food consumption among teenagers. Teenagers from census tracts with SNAP–Ed interventions may have opted to use their disposable income on fast food in direct response to more healthful snacks and meals being offered at home that resulted from SNAP–Ed interventions directed to their parents. Alternatively, teenagers in the intervention census tracts may attend schools with nearby fast food restaurants.

Teenagers in low-reach census tracts had higher levels of physical activity than teenagers in no-intervention census tracts. Limited statistical power may be responsible for the lack of significant findings for teenagers in moderate- and high-reach areas.

One advantage of this study is that all census tracts from which EARS and CHIS data were obtained met the same criteria for SNAP–Ed eligibility. Nonsignificant differences between intervention groups (no vs low, moderate, or high) compared by age, sex, race/ethnicity, education, and FPL strengthen the case that SNAP–Ed interventions may explain more healthful behaviors among adults and children. However, this study is limited in that we do not know the extent to which CHIS participants in the low-, moderate-, or high-reach groups actually participated in an intervention; we know only that greater levels of reach heightened the probability that a CHIS respondent was also a SNAP–Ed participant. In addition, this study did not examine how the unique characteristics of the census tracts may have differed among the reach groups. The high-reach census tracts, for example, may be located in cities or counties that are more likely to have adopted policies or have environmental supports that encourage more healthful eating. Alternatively, the low-reach census tracts may have had fewer fast food establishments.

For many participants in this study, there was an established time order between presumed SNAP–Ed intervention exposure and behavior change. SNAP-Ed interventions used to calculate the independent variable (intervention reach) occurred during 8 months (October 2010 through May 2011) before the assessment of the dependent variables (June 2011 through January 2012). Moreover, CHIS was administered 4 months after FFY 2011 ended on September 30, 2011. However, the overlap in EARS and CHIS data collection subjects this study to the limitation of the cross-sectional design in establishing a true temporal relationship between the independent and dependent variables.

Finally, it is unclear whether the CHIS participants in this study were exposed to non-SNAP–Ed interventions or other factors that may have influenced their behaviors. Other organizations also target in-need populations in our high-reach census tracts to implement interventions or campaigns. The CHIS questionnaire does not ask about the Special Supplemental Nutrition Program for Women, Infants, and Children, and therefore we could not control for participation in this program in our analyses. Given these limitations, one should interpret our findings of significant relationships between SNAP–Ed interventions and more healthful dietary intake with caution.

The Healthy, Hunger-Free Kids Act of 2010 requires an annual reduction in SNAP–Ed funding by 10% so that by 2018 states will receive half of the funding that was available in FFY 2013 (11). Our study provides support to maintain and ideally expand SNAP–Ed interventions as a means to address the obesity epidemic in the United States. Currently funded SNAP-Ed contractors as well as those developing new programs should look to alternative sources of funding, and they could use our findings to justify continued or new support of direct-service interventions. Support is particularly important in California because of the state’s growing Latino population, a group that is at increased risk of obesity and its health consequences (12). Future research should address the limitations of this study, including the lack of neighborhood factors that could affect behaviors that prevent or reduce unhealthy weight gain.
